# Recombinant Alpha-1 Antitrypsin as Dry Powder for Pulmonary Administration: A Formulative Proof of Concept

**DOI:** 10.3390/pharmaceutics14122754

**Published:** 2022-12-08

**Authors:** Annalisa Bianchera, Esraa’a Alomari, Annalisa Michielon, Gianluca Bazzoli, Nicoletta Ronda, Giovanni Pighini, Ilaria Zanotti, Carmine Giorgio, Andrea Mozzarelli, Ruggero Bettini, Stefano Bruno

**Affiliations:** 1 Department of Food and Drug, University of Parma, 43124 Parma, Italy; 2 Biopharmanet-TEC, University of Parma, 43124 Parma, Italy

**Keywords:** alpha-1 antitrypsin, recombinant protein, pulmonary drug delivery, inhalable dry powder

## Abstract

Alpha-1 antitrypsin (AAT) deficiency is a genetic disorder associated with pulmonary emphysema and bronchiectasis. Its management currently consists of weekly infusions of plasma-purified human AAT, which poses several issues regarding plasma supplies, possible pathogen transmission, purification costs, and parenteral administration. Here, we investigated an alternative administration strategy for augmentation therapy by combining recombinant expression of AAT in bacteria and the production of a respirable powder by spray drying. The same formulation approach was then applied to plasma-derived AAT for comparison. Purified, active, and endotoxin-free recombinant AAT was produced at high yields and formulated using L-leucine and mannitol as excipients after identifying compromise conditions for protein activity and good aerodynamic performances. An oxygen-free atmosphere, both during formulation and powder storage, slowed down methionine-specific oxidation and AAT inactivation. This work is the first peer-reviewed report of AAT formulated as a dry powder, which could represent an alternative to current treatments.

## 1. Introduction

Human alpha-1 antitrypsin (hAAT) is a glycosylated serpin of 394 amino acids produced mainly by the liver and, to a lesser extent, by circulating monocytes and alveolar macrophages in the lungs, intestinal tissue, and kidneys [1,2]. It is one of the main protein components of plasma, with a concentration of 1.5–3.5 g/L and a circulatory half-life of 4–5 days, strongly affected by the degree of glycosylation [3,4,5]. AAT covalently and irreversibly inhibits—among other proteases—neutrophil elastase (EC 3.4.21.37), a serine protease that works both as an intracellular and extracellular microbicidal agent [6]. Hereditary mutations of the gene that encodes hAAT are responsible for alpha-1 antitrypsin deficiency (AATD, M.I.M. # 613490), a genetic disorder inherited in an autosomal codominant pattern [7,8,9]. In AATD, deficient or inactive hAAT does not counterbalance neutrophil elastase, which breaks down the lung structure through the proteolysis of elastin [10], thus inducing pulmonary emphysema and bronchiectasis. The management of AATD currently consists of augmentation therapy with weekly infusions of AAT (60 mg/kg body weight) purified from human plasma. However, weekly intravenous administrations are invasive, and plasma-purified AAT is costly, limited by blood donations, and it is prone to pathogen transmission [7]. Moreover, only 3% of infused hAAT reaches the lungs [11], where most of the damage of AATD is observed [12], whereas the liver manifestations do not benefit from it [13].

The direct administration of hAAT to the lungs has been explored to overcome some limits of intravenous infusions [14,15]. Liquid aerosols are currently under clinical investigation as a non-invasive alternative. Another way to deliver proteins to the lung is by dry powder inhalers (DPIs). Both methods have advantages and disadvantages. In the case of liquid aerosols, the penetration of the droplets in the airways, a key requirement for the therapeutic effect [16], may represent a potential issue. Moreover, during nebulization, protein therapeutics can be denatured and inactivated by oxidation or aggregation at the air–liquid interface or by mechanical stress and thermal degradation [17]. On the other hand, the production of DPIs is often carried out by processes, such as freeze drying or spray drying, which can expose the protein to thermal and physico-chemical stresses, causing a reduction of its activity. However, for chronic administration, the use of dry powder inhalers results in a better acceptance and adherence to the therapy, as well as better life quality with respect to the use of nebulizers, as the time needed for administration would be dramatically reduced compared to nebulization [15,18]. Spray drying is the technology of choice for producing inhalable powders since it allows the production of particles in the micrometer range, typically with a narrow particle size distribution. Different patents were filed in the past describing the drying of AAT by lyophilization (EP1684719; EP1685160) [19,20]. An international patent describing a method for preparing hAAT as a dry powder at high protein concentrations was granted to Nektar Therapeutics (San Francisco, CA, USA) in 1995 (EP0866726) [21]. The US version of the patent was then assigned to Novartis (Basel, Switzerland) but expired before any products reached the market. More recently, Kamada (Rehovot, Israel) filed a patent application (EP3565584) [22] detailing the preparation of an inhalable AAT powder by spray drying. Nevertheless, to our knowledge, no peer-reviewed work has been published, and no data on AAT stability and activity upon formulation are available, a piece of crucial information considering the well-known effects of spray drying on protein stability [23]. In particular, hAAT is known to undergo oxidation at a specific methionine residue, an inactivating modification also observed in cigarette smokers with AATD [24].

The administration through the lungs opens the possibility of employing recombinant AAT (rAAT), including engineered variants endowed with higher stability or improved pharmacokinetics [14]. The lack of the glycosylation pattern of plasma-derived hAAT—which could not be reproduced even in mammalian cells [14]—is most relevant to its half-life upon intravenous infusion. Indeed, it was shown that non-glycosylated rAAT is equally stable and more protective than glycosylated AAT in the respiratory tract of mice [25]. Moreover, recombinant alternatives to plasma-purified hAAT might reduce the risk of transmission of bloodborne pathogens [26] and lower production costs. To date, several host cells and expression systems have been tested [27], including bacteria [28,29,30,31,32].

Here, we developed a proof-of-concept formulation of rAAT from *Escherichia coli* as dry powder for inhalation produced by spray drying, combining the potential advantages of a recombinant source with a novel delivery mode. As the spray drying process may be a source of stress resulting in degradation or denaturation of the protein, we paid particular attention to the effect of formulation parameters on protein stability, as well as to the possible mechanisms of degradation. Finally, to investigate the possible role of glycan moieties on the formulation, the same formulation approach was applied to plasma-derived hAAT.

## 2. Materials and Methods

### 2.1. Chemicals

All chemicals and materials were purchased from Sigma-Aldrich (Milan, Italy) unless otherwise stated. Prolastin^®^ (Vicopisano, Italy) was from Grifols Italia SpA.

### 2.2. Expression Vector

The *Escherichia coli* codon-optimized human AAT gene (NCBI Gene ID: 5265) fused with an N-terminal hexa-His tag followed by a recognition site for the TEV protease was produced by GeneArt (Thermo Fisher Scientific Italia, Rodano, Italy) and subcloned in a pET-28a vector between restriction sites NcoI and BamHIII (pET28_6His-AAT). The sequence of the gene was confirmed by sequencing. *E. coli* BL21(DE3) cells were transformed with the vector by electroporation, and a glycerol stock was stored at −80 °C.

The resulting protein sequence was:

MGSSHHHHHHENLYFQGEDPQGDAAQKTDTSHHDQDHPTFNKITPNLAEFAFSLYRQLAHQSNSTNIFFSPVSIATAFAMLSLGTKADTHDEILEGLNFNLTEIPEAQIHEGFQELLRTLNQPDSQLQLTTGNGLFLSEGLKLVDKFLEDVKKLYHSEAFTVNFGDTEEAKKQINDYVEKGTQGKIVDLVKELDRDTVFALVNYIFFKGKWERPFEVKDTEEEDFHVDQVTTVKVPMMKRLGMFNIQHCKKLSSWVLLMKYLGNATAIFFLPDEGKLQHLENELTHDIITKFLENEDRRSASLHLPKLSITGTYDLKSVLGQLGITKVFSNGADLSGVTEEAPLKLSKAVHKAVLTIDEKGTEAAGAMFLEAIPMSIPPEVKFNKPFVFLMIEQNTKSPLFMGKVVNPTQK

### 2.3. Culture Media

– LB medium: 10 g/L tryptone, 5 g/L yeast extract, 10 g/L NaCl.
– Trace elements solution: 6 g/L CaCl_2_ 2 H_2_O, 6 g/L FeSO_4_ 7H_2_O, 1.15 g/L MnCl_2_ 4 H_2_O, 0.8 g/L CoCl_2_ 6H_2_O, 0.7 g/L ZnSO_4_ 7H_2_O, 0.3 g/L CuCl_2_ 2H_2_O, 0.02 g/L H_3_BO_3_, 0.25 g/L (NH_4_)_6_Mo_7_O_24_ 4H_2_O, 5 g/L ethylenediaminetetraacetic acid (EDTA). The components were dissolved in the minimum amount of HCl and added to water containing EDTA. The solution was autoclaved and stored in the dark at 4 °C.
– MgCl_2_ solution: 1 M MgCl_2_, filter-sterilized.
– HCDC medium [33]: 10 mL/L of the trace element solution, 10 mL/L of the MgCl_2_ solution, 2 g/L yeast extract, 10 mL/L glycerol, 10 g/L K_2_HPO_4_, 10 g/L KH_2_PO_4_, 91 g/L Na_2_HPO_4_ 2H_2_O, 1.1 g/L NH_4_Cl, 3.7 g/L KCl, 6.6 g (NH_4_)_2_SO_4_. The medium (7 L) was heat-sterilized inside the bioreactor.

### 2.4. Shake-Flask Experiments

Preliminary expression trials in shake-flask experiments were carried out to determine the best expression conditions before the scaling-up. Single colonies of transformed BL21(DE3) *E. coli* cells were inoculated in 5 mL LB medium and grown overnight. The culture was then inoculated in 1 L LB medium in a 5 L flask. Kanamycin was added at a final concentration of 50 µg/mL to all media.

### 2.5. Fed-Batch Culture

To increase cell density and, therefore, the yield of recombinant AAT, a fed-batch culture approach with pH, oxygenation, and nutrient control was pursued [34]. A 5 mL overnight culture of *E. coli* transformed with the pET28_6His-AAT plasmid was inoculated in 50 mL of LB medium added of 50 µg/mL kanamycin in a 250 mL flask and incubated overnight at 37 °C with shaking at 220 rpm. The fresh culture was inoculated in 6 L HCDC medium in a Sartorius Biostat^®^C plus bioreactor (Sartorius Italia, Varedo, Italy) equipped with pH, foam, temperature, and pO_2_ probes and three peristaltic pumps for the controlled addition of alkali, antifoam, and feeding solutions. During growth, the HCDC medium was supplemented with a concentrated nutrient solution containing 70 mL/L of the trace element solution, 15 mL/L of the MgCl_2_ solution, and 70% (*v*/*v*) glycerol, following an increasing feeding profile (0.25 mL/min from 2 to 5 h of growth, 0.5 mL/min from 5 to 10 h, 0.75 mL/min from 10 to 15 h). A 20% antifoam solution was added as feedback from the high-foam sensor. Cultivation was carried out under controlled conditions at 37 °C and pH 6.8. The pH was kept constant by the controlled addition of 20% NH_4_OH. The dissolved oxygen concentration (DO) was monitored using pO_2_ electrodes (Mettler Toledo, Switzerland) and maintained at 30% saturation (with 100% corresponding to air) by increasing agitation speed in the 20–1000 rpm range with a constant airflow of 10 mL/min. After 10 h, the temperature was lowered to 20 °C, and rAAT expression was induced by the addition of filter-sterilized 2 mM isopropyl β-D-thiogalactoside (IPTG). Thirteen hours after induction, cells (143 g bio wet weight per liter) were harvested by centrifugation using an Eppendorf 5920 centrifuge cooled at 4 °C. The cell paste was washed three times with an 8 mM Tris-HCl solution at pH 8.0 and then stored at −80 °C.

### 2.6. Protein Purification

The cell paste was thawed and suspended in three volumes of a solution containing 20 mM KH_2_PO_4_, 0.2 M NaCl, 0.2 mM PMSF, 0.2 mM benzamidine, and 1 mM pepstatin, at pH 8. Cells were then broken using a Panda Plus 200 homogenizer (G.E.A., Parma, Italy) in a single cycle at 500 torr. Protein purification was carried out by fractional separation with ammonium sulfate followed by IMAC chromatography [35]. The cell lysate was centrifuged for 45 min at 20,000× *g* at 4 °C and 35% (NH_4_)_2_SO_4_ was added to the supernatant to promote precipitation of the less soluble contaminants. Upon centrifugation, the supernatant was further added to (NH_4_)_2_SO_4_ to 75% final concentration to produce AAT precipitation. The pellet—containing partially purified rAAT—was resuspended with 50 mL volumes of a solution containing 20 mM Tris, 0.5 NaCl, and 5 mM imidazole, at pH 8. The resulting solution was incubated with 5 mL of Ni Sepharose 6 fast flow slurry (Merck, Darmstadt, Germany) previously washed with 2% NaOH to remove lipopolysaccharides (LPSs) and equilibrated with 5 volumes of the loading solution. The slurry was extensively washed in batches with a solution containing 20 mM Tris, 0.5 M NaCl, and 20 mM imidazole at pH 8. Finally, the protein was eluted with 50 mL of a solution containing 20 mM Tris, 0.5 NaCl, and 250 mM imidazole at pH 8. EDTA at 1 mM final concentration was added to the protein solution immediately after elution. The eluted protein was collected and dialyzed against PBS (137 mM NaCl, 10 mM phosphate, and 2.7 mM KCl, at pH 7.4) at 4 °C overnight and flash frozen in 10 mL aliquots. Purity and yield were assessed by Coomassie blue-stained SDS-PAGE.

### 2.7. Endotoxin Removal and Evaluation of the Proinflammatory Activity of rAAT Solutions

In view of in vivo experiments, the protein solution was sterilized using 0.2 µm filters and passed 3 times through Sartobind Q STIC PA endotoxin removal capsules (Sartorius Italia, Varedo, Italy), as per the manufacturer’s instructions. The efficacy of endotoxin removal and the actual absence of proinflammatory activity in the final preparation were assessed by a functional assay on the cultured human monocyte cell line THP-1 (Sigma-Aldrich, Milan, Italy). THP-1 cells were cultured in 12-well plates in the presence of 50 ng/mL of phorbol 12-myristate 13-acetate (PMA; Sigma-Aldrich, Schnelldorf, Germany) for 72 h to allow the cells to differentiate into macrophages. Cells were then incubated for 24 h with cell culture medium alone or with medium containing lipopolysaccharide 1 μg/mL, or rAAT 1.8 μg/mL submitted or not to endotoxin removal, or PBS used as a vehicle for rAAT. Interleukin-6 (IL-6) was measured in the macrophage supernatants with a commercially available ELISA kit (Thermo Scientific, Waltham, MA, USA).

### 2.8. Activity Assays

AAT activity was tested using a chromogenic elastase inhibition assay [36] using the non-natural substrate N-succinyl-Ala-Ala-Ala-p-nitroanilide to measure the residual activity of elastase upon incubation with AAT. Reduction in elastase activity is proportional to AAT activity. Briefly, rAAT was incubated at 5 nM concentration, as determined by SDS-PAGE, with 10 nM human or porcine elastase in a solution containing 0.1 M HEPES, 0.5 M NaCl, and 0.05% Triton, pH 7.4 at 37 °C for 45 min. The residual elastase activity was determined by following the hydrolysis of N-succinyl-Ala-Ala-Ala-p-nitroanilide to p-nitroaniline at 410 nm in a Cary 4000 UV–Vis spectrophotometer (Varian). As elastase concentration was double the concentration of rAAT in the incubation mixture, the fraction of active rAAT with respect to the amount estimated by SDS-PAGE was calculated as the complement of the fractional residual activity of elastase in comparison to non-inhibited elastase. To assess the stability of the protein in PBS solution, aliquots were stored at −80 °C, −20 °C, +4 °C, and +25 °C, and their activity was followed over time. The degree of protein precipitation was assessed by SDS-PAGE. All measurements were carried out in at least two replicates.

### 2.9. SEC Chromatography

Analytical size exclusion chromatography (SEC) allows estimating the molecular weight of proteins under non-denaturing conditions, thus helping elucidate their oligomeric state and the presence of aggregates. A Shimadzu HPLC system (Prominence) coupled with a UV detector and LabSolutions software (Shimadzu, Kyoto, Japan) was employed. Chromatographic separation was achieved on a Phenomenex BioSep-2000 (300 mm, 150 mm, 5 μM) in an isocratic elution mode. The column was pre-equilibrated and then developed with a solution containing 50 mM K_2_HPO_4_, and 300 mM NaCl pH 7 at a flow rate of 1 mL/min. The manual injection volume was 50 μL and the protein concentration was 1 mg/mL. Detection was performed by the SPD-20A Model UV detector at 220 nm. The total chromatographic run period was 15 min. The experiments were carried out at room temperature. The column was calibrated with myoglobin (17 kDa), ovalbumin (44 kDa), IgG (150 kDa), IgA (300 kDa), and bovine thyroglobulin (670 kDa). The sample was centrifugated before loading.

### 2.10. Circular Dichroism

Circular dichroism (CD) spectra were collected with a Jasco J-1500 spectropolarimeter equipped with a Peltier thermostatic unit set at 20 °C and using 0.1 mm quartz cells. The software was JASCO Spectra Manager II. The protein concentration was 5 µM in buffer 10 mM K_2_HPO_4_ at pH 7. The spectral scans were collected between 250 and 180 nm, 0.5 nm data pitch, 8 s DIT, 2 nm bandwidth, at 50 nm/min scanning speed. Each spectrum is the result of 3 averaged accumulations. Secondary structure estimation was performed by using the Dichroweb server. All CD spectra were corrected for buffer background. Far-UV CD signal changes at 220 nm were monitored as a function of increasing temperature from 20 to 90 °C, with steps of 5 °C and with an equilibration time of 1 s at each temperature before recording a reading. The thermal transitions were analyzed with CalFitter software online.

### 2.11. Evaluation of Oxidation by Mass Spectrometry

rAAT, either from solutions or resolubilized from powders, was separated by SDS-PAGE. The bands corresponding to rAAT were excised, incubated with a solution containing 50% ethanol and 10% acetic acid until fully destained, washed twice with a buffered solution containing 25 mM ammonium bicarbonate and pure acetonitrile (ACN) 1:1 for 20 min, and finally incubated with pure ACN for 5 min to complete spot dehydration. After the removal of ACN, a solution containing trypsin in a 25 mM ammonium bicarbonate solution, pH 7.4, was added for gel rehydration. In-gel digestion was performed at 37 °C for 16 h. The reaction of trypsin was stopped by the addition of ACN: 0.1% trifluoroacetic acid (TFA) 1:1. Peptides were extracted by incubating the gel fragment with ACN: 0.1% TFA 1:1 twice for 20 min at 37 °C, before complete drying using a vacuum concentrator and resuspension with ACN:TFA 0.1% 1:1 before mass spectrometry experiments. Mass spectrometry on digested peptides was carried out using an LTQ Orbitrap (Thermo Fisher Scientific, Waltham, MA, USA) mass spectrometer. The peptide mixture was separated in a Phenomenex Aeris™ PEPTIDE 3.6 µm XB-C18 (150 mm × 2.1 mm) reverse-phase column, developed in a 0.2% formic acid/water−0.2% formic acid/acetonitrile gradient (200 μL/min). Peptide identification from LTQ Orbitrap experiments was carried out using the software PEAKS Studio (version 8.5, Bioinformatics Solutions, Waterloo, ON, Canada), set to a precursor mass tolerance of 10 ppm and a fragment mass error tolerance of 0.2 Da.

### 2.12. Set Up of Spray-Drying Conditions

To preliminarily screen the most promising conditions for the spray drying of the protein, different setups, buffers, and bulking agents were evaluated without the protein. The amino acids L-cysteine, L-lysine, or L-leucine (all from Sigma-Aldrich^®^, Schnelldorf, Germany) were added as lubricant agents to increase the yield of recovery and to prevent moisture [37,38]. Moreover, trehalose (A.C.E.F., Fiorenzuola, Italy), sucrose (A.C.E.F., Fiorenzuola, Italy), and mannitol (Pearlitol^®^ SD200, Roquette, France) were assayed at a concentration of 0.5 mg/mL as bulking agents and protein stabilizers and compared for their contribution to yield and flow characteristics of the spray-dried powder [39]. The solutions were spray-dried with a Mini Spray Dryer B-290 (BÜCHI Labortechnick, Flawil, Switzerland) equipped with a spray nozzle having a diameter of 0.5 or 0.7 mm. Parameters were set as follows: inlet temperature (110 °C), drying gas flow rate (742 L/h), aspiration (30 m^3^/h), and solution feed rate (1.5 mL/min).

After having identified sucrose, mannitol, and leucine as potential excipients, a 2^3^ full factorial design of experiment was set up to evaluate the effect of variables such as type of bulking agent, nozzle diameter, and leucine concentration on the efficiency of the process of spray drying without the protein. The levels and coding of variables of the experimental design are reported in Table 1. Selected outputs were the yield, the emitted fraction (defined as the quantity of powder emitted from the device with respect to loaded quantity), and the fine particle fraction, which expresses the percentage of the emitted mass contained in an aerosol that is small enough to enter the deep lungs (particles < 5 µm in aerodynamic diameter). Emitted fraction and fine particle fraction were assessed by fast screening impactor analyses (see below).

### 2.13. Protein Formulation

In preparation for spray drying, the protein solution was dialyzed against a potassium phosphate buffer solution (K_2_HPO_4_-KH_2_PO_4_). The most promising spray-drying conditions identified in the preliminary tests were then applied. Protein activity was checked at each step by resuspending the powders in PBS buffer, quantifying the soluble protein by SDS-PAGE, and carrying out the antielastase enzyme assay. To prevent oxidation, the protein solution was spray-dried using nitrogen as nebulizing gas. The outlet temperature was checked for the whole duration of drying and was recorded as 65 °C.

### 2.14. Thermal Analysis of Spray-Dried Powder

Dried powders were analyzed by differential scan calorimetry (DSC) with a DSC Mettler821e driven by STARe software (Mettler Toledo, Columbus, OH, USA). About 5 mg of dried formulation was put into a 40 µL aluminum crucible (ME-27331, Mettler Toledo) which was sealed and pierced. Scans were performed under a flux of dried nitrogen (100 mL/min) between the range 25 and 280 °C at a heating rate of 10 °C min^−1^. Thermogravimetric analysis was performed in a TGA/DSC1 operated by STARe software (Mettler Toledo, Columbus, OH, USA). Alumina pans (volume 100 µL) were filled with about 5 g of powder and heated at a rate of 10 °C from 25 °C to 125 °C under a flux of dried nitrogen of 80 mL/min.

### 2.15. Flowability of Powders

The flow characteristics of powders were characterized by analyzing the dynamic angle of repose, as reported in European Pharmacopeia (10th edition). The powder was put in a 10 mL glass vial accommodated in a friability tester (model TA3R, Erweka GmbH, Langen, Germany), from which the drum had been removed so that the bottom of the vial was facing the observer. The tester was activated to rotate the vial at 20 rpm for a minute: a video of the vial was recorded and at least 6 frames were analyzed employing the software Image J64 (NIH, Bethesda, MD, USA) to determine the dynamic angle of repose of powders.

### 2.16. Analysis of Particle Size Distribution

Spray-dried powders were characterized in terms of particle size distribution by a laser light scattering technique (Spraytec^®^, Malvern Instruments Ltd., Malvern, UK). The diffractometer was equipped with a 300 mm focal lens that can measure particles in a size range between 0.1 and 900 µm. Samples were prepared by dispersing 10 mg of powder in 10 mL of 0.1% *w*/*v* Span 85 (Honeywell Fluka^TM^, Morris Plains, NJ, USA) in cyclohexane (Sigma-Aldrich^®^, Schnelldorf, Germany). Each sample was sonicated in an ultrasonic bath (Branson Ultrasonics Corporation 8510, Brookfield, CT, USA) for 5 min right before the analysis to help particle separation and improve homogeneity. Each measure was performed in triplicate. A cumulative size distribution curve was obtained and described in terms of volume diameter of the 10th (D_v,10_), 50th (D_v,50_), and 90th (D_v,90_) percentile of the particle population. The amplitude of the distribution was described by span value, calculated as [(D_v,90_ − D_v,10_)/D_v,50_].

### 2.17. In Vitro Assessment of Aerodynamic Performance

The aerodynamic performance of powders aerosolized from a capsule in a dry powder inhaler (DPI) was assessed using a fast screening impactor (FSI, Copley Scientific Ltd., Colwick, UK). Hypromellose capsules (Qualicaps^®^ V-I, size 3, Whitsett, NC, USA) were filled with 10 mg of powder and inserted in a mid–high-resistance DPI (RS01 Plastiape, Osnago, Italy) for aerosolization. The accurate amount of powder in each capsule was recorded. The impactor was connected to a vacuum pump (Mod. 1000, Erweka GmbH, Langen, Germany) to generate the airflow to aerosolize the powder: the flow rate was controlled by a TPK Critical Flow Controller (Copley Scientific Ltd., Colwick, UK) at 60 L/min for 4 s, to produce a pressure drop of 4 kPa over the inhaler. DPIs were attached employing a mouthpiece adaptor to the induction port of the impactor and the pump actuated. The fraction of powder with aerodynamic diameter below 5 µm, corresponding to the fine particle dose, was determined by weighing the amount of powder collected on the filter (glass filter type A/E, diameter 76 mm, Pall Corporation, New York, NY, USA) in the fine fraction collector and compared to the amount of powder emitted by the aerosolization process or loaded in the capsule, to calculate emitted fraction (EF%) and fine particle fraction (FPF%), respectively. A single capsule was discharged for each test, which was repeated three times.

### 2.18. Scanning Electron Microscopy (SEM)

The morphology of spray-dried formulations was investigated by a scanning electron microscope (FESEM-FIB Zeiss Auriga Compact, Darmstadt, Germany). Samples were prepared by depositing powder on adhesive carbon tapes mounted on aluminum stubs and by removing excess powder with a gentle nitrogen flow. High vacuum conditions (1.87 × 10^−4^ Pa) were reached by depressurization and images were taken with an accelerating voltage of 1.0 kV and a working distance of 4.9 mm.

### 2.19. Assessment of AAT Content in the Powder

The quantity of AAT in the powder was determined for every formulation by SDS-PAGE of a solution obtained by dissolving the powder in PBS. The stability of the formulation was assessed by measuring the residual activity of AAT over several months.

### 2.20. Dynamic Light Scattering (DLS)

The starting rAAT solution and solution resulting from redissolution of spray-dried powders were analyzed by dynamic light scattering. Measurements were performed with a Zetasizer Nano (Malvern Instruments, Malvern, UK) equipped with a 633 nm laser, using NIBS detection (173° backscatter) at 25 °C. The buffered starting solution of the recombinant protein was diluted 1:10 with ultrapure water to obtain a protein concentration of 0.15 mg/mL. The formulated powders were redissolved in ultrapure water at a concentration of 0.3 mg/mL. Three measurements were performed for each sample and used to calculate the average hydrodynamic diameter by number.

## 3. Results

### 3.1. Expression and Purification of rAAT

**Figure 1:**
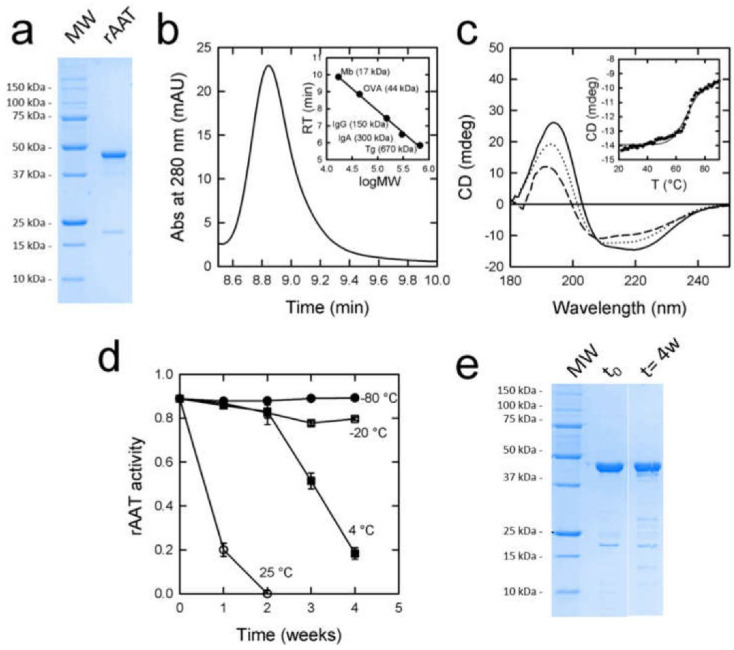
(**a**) Coomassie blue-stained SDS-PAGE gels of purified rAAT. (**b**) SEC chromatogram and calibration curve (inset). (**c**) Circular dichroism spectra of rAAT before heating (solid line), upon incubation at 90 °C (dashed line), and after a slow return to 20 °C (dotted line). Inset: Thermal ramp experiment between 20 and 90 °C; the black line is the fitting obtained with the CalFitter algorithm. (**d**) Antielastase activity of rAAT sampled over time upon storage at −80 °C, −20 °C, 4 °C, and 25 °C. Data are shown as mean ± SE. (**e**) SDS-PAGE of rAAT solution before (t0) and after incubation at 4 °C for 4 weeks (t = 4 w).

*E. coli* BL21 cells transformed with a pET28-derived plasmid encoding His-tagged AAT were grown in a Sartorius Biostat^®^C plus bioreactor, yielding an average of 1 kg of cell paste from 7 L of culture. The fractional precipitation step of the cell lysate with ammonium sulfate led to the recovery of approximately 95% of rAAT, with a purity of around 60% based on the densitometric analysis of SDS-PAGE electropherograms. IMAC chromatography applied to the partially purified protein led to a final purity of around 93%, as assessed by SDS-PAGE (Figure 1a). The densitometric analysis of the prevalent band in comparison with the molecular weight markers led to an estimation of the MW of 47 kDa, consistent with the theoretical value of 46.361 kDa for rAAT. An unknown contaminant accounting for 5% of the total protein content was observed at 17 kDa even after purification with ammonium sulfate and IMAC chromatography. The amount of AAT in all experiments was measured by densitometric analysis of the AAT band of SDS-PAGE to avoid the contaminant contribution. Overall, an average of 400 mg of purified rAAT was obtained from a 7 L culture. rAAT was dialyzed against a solution (PBS) containing 137 mM NaCl, 2.7 mM KCl, 10 mM Na_2_HPO_4_, 1.8 mM KH_2_PO_4_, at pH 7.4, and flash frozen.

### 3.2. Endotoxin Removal

With the prospect of administering the powder in vivo, endotoxins were removed from rAAT using Sartobind Q STIC PA capsules. The rAAT solution upon purification strongly stimulated macrophage IL-6 secretion, an index of proinflammatory activity (Figure S1). A lipopolysaccharide solution (LPS CTRL) was used to stimulate cells, as a positive control. The PBS solution used for rAAT dilution (PBS) was also tested. No IL-6 was detected in cell supernatants upon incubation with either PBS or powder rAAT preparations after endotoxin removal. Therefore, upon endotoxin removal, the rAAT solution proinflammatory activity, evaluated as IL-6 secretion, was abolished.

### 3.3. rATT Characterization

The antielastase activity of rAAT was 93%, as calculated from the ratio between the observed activity—assessed through the elastase inhibition assay—and the protein concentration—evaluated by SDS-PAGE.

The densitometric analysis of SDS-PAGE gels led to estimates of a molecular mass of 44 kDa and 51.7 kDa for rAAT (Figure 1a) and (glycosylated) hAAT (Figure S2a in the Supplementary Material), respectively, in agreement with the expected masses of 46.36 kDa and 51 kDa. Size exclusion chromatography (SEC) yielded an estimated molecular mass of 42.6 kDa for rAAT (Figure 1b), confirming the monomeric state of the protein. For comparison, hAAT exhibited an apparent molecular mass of 68.25 kDa (Figure S2b in the Supplementary Material), evidently because of the effect of glycosylation on SEC separation.

The circular dichroism spectrum of rAAT (Figure 1c) was analyzed with the Dichroweb algorithm for secondary structure estimation [40], resulting in 21.4% alpha-helices, 31.3% beta-sheets, and 26.4% of non-ordered structures, in good agreement with the analysis of the hAAT spectrum (Figure S2c in the Supplementary Material), which yielded 28.4% alpha-helices, 27% beta-sheets, and 19.7% non-ordered structures.

### 3.4. rATT Stability in Solution

To assess the stability of rAAT, a continuous temperature ramp experiment was performed by monitoring the circular dichroism signal at 220 nm. The resulting curves indicated a transition at 68 °C (Figure 1c, inset), identical to that measured for hAAT (Figure S2c, inset in the Supplementary Material). The analysis of the spectrum recorded at 90 °C (upper instrumental limit) indicated that rAAT maintains a native-like structure, with a small decrease in alpha-helices (to 20.8%) and an increase in non-ordered structures (to 29.7%). A slow return to 20 °C led to a spectrum (Figure 1c) whose analysis indicated a secondary structure composition closer to rAAT before heating (30.1% alpha-helices, 30.7% beta-sheets, 28.2% non-ordered structures).

rAAT activity was checked over time by monitoring the elastase-inhibition activity upon storage at different temperatures under a non-controlled atmosphere. Flash-frozen rAAT stored at −80 °C and −20 °C maintained full activity over several weeks (Figure 1d). rAAT solutions stored at 4 °C lost 50% of the activity in three weeks, whereas solutions stored at 25 °C completely lost their activity within a few days (Figure 1d). The SDS-PAGE of samples stored for 4 weeks at 4 °C showed that the protein remained soluble and mostly intact (Figure 1e), hinting at inactivation through oxidation rather than aggregation or proteolysis.

To identify the mechanism of the observed activity loss of rAAT when stored at room temperature, we assessed by LC-MS the modifications of Met358 and Met351, which are known to oxidize irreversibly to form methionine sulfoxide [41,42]. rAAT stored under different conditions was digested with trypsin, and the peptide KGTEAAGAMFLEAIPMSIPPEVKF, which contains both Met358 and Met351, was monitored by LC-MS. A molecular weight of 2259.03 Da was observed for the freshly prepared protein. rAAT incubated in PBS for 7 days at 25 °C exhibited the peptide in the non-oxidized form (2259.03 Da), in a mono-oxidized form (+16 Da), and in a di-oxidized form (+32 Da), whose AUCs in the chromatogram accounted for 57.4%, 34.3%, and 8.3% of the total signal, respectively (Figure S3 in Supplementary Material). Therefore, the slow activity loss could be attributed to the progressive mono- and di-oxidation of Met358 and Met351.

### 3.5. Formulation of Recombinant rAAT as Dry Powder

#### 3.5.1. Effect of Buffering Agents and Excipients

Since protein formulations are bound to the medium in which proteins are stable, within a limited range of pH, we first evaluated spray drying of rAAT in different buffered solutions that were preliminarily shown to be compatible with rAAT stability, i.e., TRIS buffer and phosphate buffer at pH 7.0–7.4. When TRIS and PBS were used, the spray-dried powder was very hygroscopic, sticky, and did not separate in the cyclone of the spray drying equipment. Thus, no powder could be recovered in the collector. In the attempt to reduce hygroscopicity, the PBS solution was modified by eliminating sodium chloride and by decreasing the concentration of phosphates to 10 mM, which was shown to be the minimum concentration of phosphate salts that promote the solubility of rAAT (Figure S4 in Supplementary Material). The resulting powder could be efficiently recovered in the collecting compartment.

Once the optimal buffer for rAAT spray drying was established, i.e., 10 mM KH_2_PO_4_-K_2_HPO_4_, pH 7.0, three non-reducing excipients, i.e., trehalose, mannitol, and sucrose, were tested in the optimized PBS solution in the absence of protein. The nozzles were set at 0.5 mm or 0.7 mm in diameter, and the inlet temperature was set at 110 °C. Based on these preliminary experiments, trehalose was excluded, as it produced a very sticky powder.

Mannitol and sucrose were further tested in association with L-leucine, L-cysteine, or L-lysine, once again in the absence of protein. No powder could be recovered in the presence of L-lysine, nor in the presence of an association of L-lysine and L-cysteine in a 1:1 weight ratio. L-cysteine alone, which was also tested because of its antioxidant properties [43], led to a relatively low yield (51%) when combined with sucrose. Moreover, the weight of the powder recovered from the association of L-cysteine with mannitol was higher than the overall mass of solids, indicating high residual water content. L-cysteine was therefore excluded from the following tests.

L-leucine, which is widely used as an excipient for spray drying of proteins [37,38] was the most promising excipient in preliminary experiments. Therefore, a 2^3^ full factorial design of experiments was set up to evaluate its concentration, its association with bulking agents, and the nozzle diameter. The concentration of either sucrose or mannitol was set at 0.5 mg/mL, and drying was performed at 110 °C. The aerodynamic performances, namely the emitted fraction and the fine particle fraction, and the technological characteristics of resulting powders, such as dynamic angle of repose and median volume diameter (Dv50), are reported in Table 2, along with the relevant combination of input parameters. The yield was calculated as the percentage of the ratio between the mass of the recovered powder and that of the solute in solution, whereas the emitted fraction and the fine particle fraction were assessed by fast screening impactor analyses. As can be observed in Table 2, all formulations provided very good results both in terms of emitted fraction and fine particle fraction.

A linear model was applied for each outcome. Only the models for yield and fine particle fraction led to statistically significant coefficients.

Yield (%) = 66 + 3.61x_1_ + 2.217x_2_ + 7.545x_3_(*) (1)

FPF (%) = 79.7 + 1.5625x_1_ + 1.1865x_2_ + 8.7875x_3_(*) (2)

The only variable significantly affecting these critical parameters was the nozzle diameter (x_3_) since, as expected, a nozzle with a larger diameter (0.7 mm) resulted in a higher yield (*p* = 0.0317), while the nozzle with a lower diameter afforded a larger fine particle fraction (*p* = 0.0126). Although not statistically significant, the coefficient related to the type of bulking agent had a positive sign, suggesting that mannitol (coded as +1) is preferable to sucrose in improving the yield.

The results on the fine particle fraction are consistent with particle size distribution analysis by laser light scattering (Table 3): for sucrose-based formulations, the effect of the nozzle diameter was more evident than for mannitol-based formulations, while the type of bulking agent and the amount of L-leucine did not significantly affect the particle size distribution. None of the remaining parameters produced significant effects on the flowability and aerodynamic characteristics of the powder (Table 2). Independently of formulation type or condition of drying, all powders showed a volume distribution with a Dv_50_ below 5 µm, and a Dv_90_ below 10 µm, suggesting that good aerodynamic performances could be expected from all combinations of excipients.

The thermal analysis of sprayed powder by DSC revealed no endothermic peaks, neither for mannitol-based nor sucrose-based formulations, suggesting that they were in amorphous form. No peaks attributable to L-leucine were identified.

Thermogravimetric analysis revealed that no residual water was present in powders obtained by the spray drying of mannitol-based formulations. On the other hand, powders obtained from sucrose-based solutions showed a small residual content of water ranging between 0.5% and 1.6%.

#### 3.5.2. Formulation of rAAT

After the preliminary characterization of the spray-dried powder containing only excipients, the most promising combinations, i.e., 0.5 mg/mL L-leucine, 0.5 mg/mL mannitol, or sucrose in 10 mM phosphate buffer, pH 7.0, were assayed in the presence of 0.5 mg/mL rAAT. The concentration of rAAT was chosen to keep a relative weight ratio of sugar to protein of 1:1 since this is the lowest amount of sugar reported to provide adequate stability to proteins [39,44]. Spray drying was performed at 110 °C, and, in consideration of the results on protein-free samples, a nozzle of 0.7 mm was chosen. Drying was performed using nitrogen as feeding gas to prevent or at least limit the oxidation of the protein during the process. For the same reason, the powder was collected under a glovebox saturated with nitrogen and stored in airtight containers.

The aerodynamic performances of the resulting powders are reported in Table 4. Unexpectedly, despite the increase in the concentration of solutes and the use of the nozzle with a larger diameter, the recovery of powder was lower than the corresponding counterparts with excipients only. This might be in part explained by the low volume of solutions that were dried (50 mL); in fact, by increasing the starting volume of the solution to 150 mL, the yield increased to 69%. As far as aerodynamic performance was concerned, the emitted fraction was significantly higher when mannitol was used as a bulking agent and comparable to that obtained with the powder containing only excipients (see Table 2), whereas the fine particle fraction was around 40% with both bulking agents. In general, the aerodynamic behavior of the powder worsened with the addition of rAAT.

**Figure 2:**
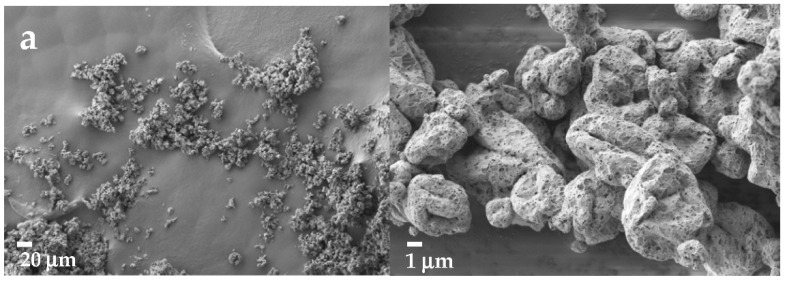

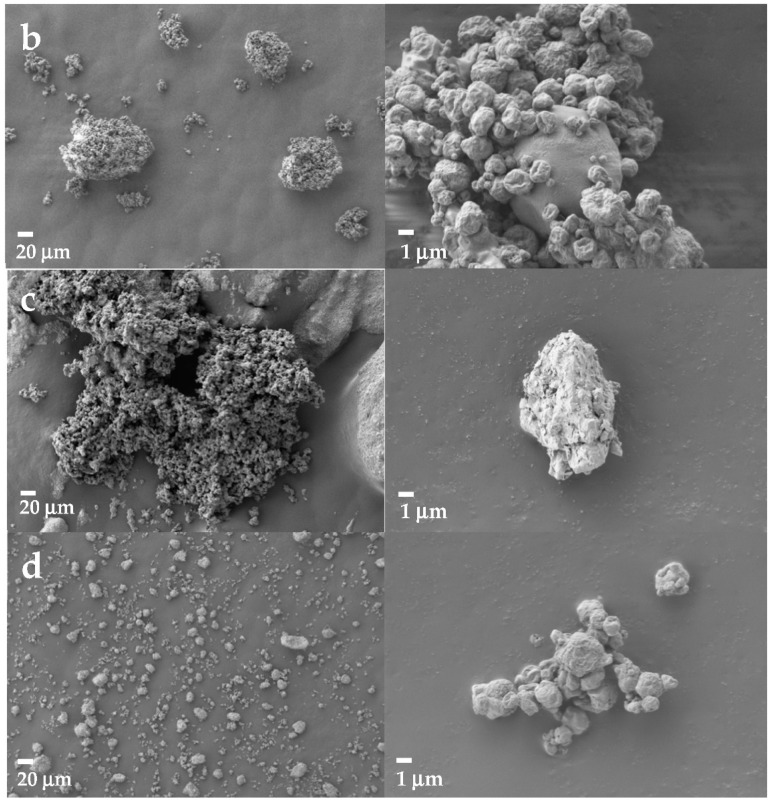
SEM images of (**a**) sucrose-based powders without rAAT; (**b**) sucrose-based powders with AAT; (**c**) mannitol-based powders without rAAT; (**d**) mannitol-based powders with rAAT. Left column, magnification 500×, scale bar 20 µm; right column, magnification 10,000×, scale bar 1 µm.

To investigate the behavior of the powders containing sucrose and mannitol, SEM analysis was performed. As shown in Figure 2a, sucrose-based powders prepared without protein exhibited a homogeneous size distribution with particles with a diameter of about 3 μm, in agreement with results derived from laser diffraction analysis. These particles were well separated or loosely aggregated. When rAAT was added to the spray-dried solution (Figure 2b), larger aggregates were obtained, composed of slightly wrinkled particles of about 1–3 μm in diameter, adhering to bigger ones. Interparticle spaces were scarce, suggestive of a stronger cohesive force among them or a partial fusion that is probably responsible for their poor aerodynamic behavior. If particles were prepared from mannitol solutions (Figure 2c), aggregates of small, fused particles were obtained. The addition of rAAT to the solution (Figure 2d) led to a fine powder, mainly composed of slightly wrinkled particles of about 2 μm in diameter, individual or in loose aggregates showing an overall size of 10 μm.

#### 3.5.3. Protein Stability and Activity upon Formulation

Upon resuspension of the powders in a PBS buffer, the protein was fully soluble (Table 4), suggesting that no aggregation occurred, neither with sucrose nor mannitol as bulking agent. The elastase inhibition activity of rAAT upon formulation was then assayed. Powders obtained with sucrose as bulking agent exhibited a drop in the activity (to 18%), which was better preserved when mannitol was used (54%) (Table 4). Based on these observations on powder aerodynamic properties and stability, the subsequent experiments were carried out with mannitol as a bulking agent. The formulation was coded as SD-rAAT.

**Figure 3:**
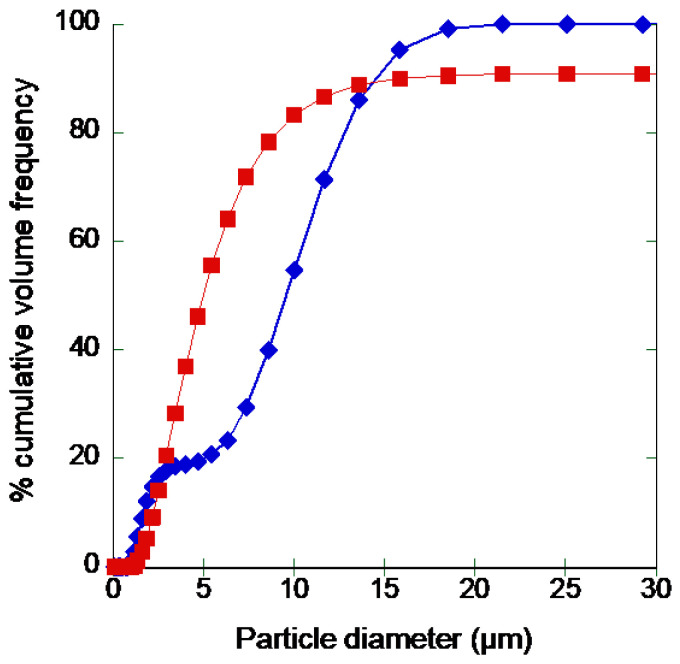
Particle size distribution analysis of SD-rAAT right after preparation (blue diamonds) and after 2 weeks of storage at −80 °C (red squares).

A common issue during the storage of powders is their tendency to aggregate due to the formation of interactions and water absorption. For this reason, SD-rAAT was checked for stability over time. The particle size distribution was evaluated by laser diffraction immediately upon producing the powder, and upon 2 weeks of storage at −80 °C (Figure 3). The size distribution of the powder after preparation is consistent with images obtained by SEM, showing the coexistence of small particles or aggregates of larger size. After freezing, apart from the presence of a few large particles, the powder preserved promising characteristics in view of aerosolization, with a Dv_50_ of 4.94 μm.

**Figure 4:**
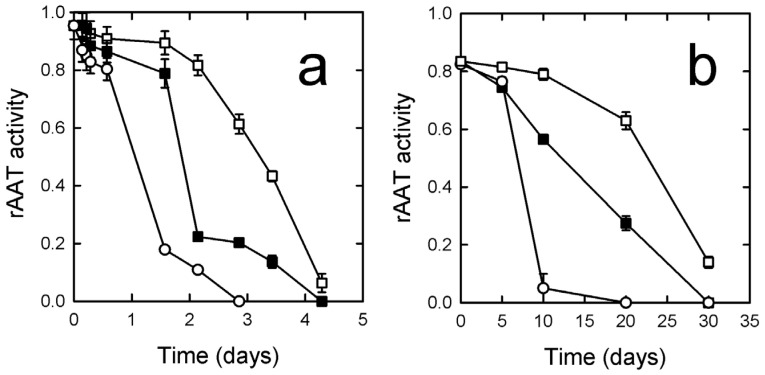
Stability of SD-rAAT powder stored in air (**a**) and oxygen-free atmosphere (**b**) at −20 °C (open squares), 4 °C (closed squares), and 25 °C (open circles). Data are shown as mean ± SE.

The activity of SD-rAAT was also followed over an extended time upon storage at different temperatures in air or under an oxygen-free atmosphere, namely in conditions that are customary in the pharmaceutical field. When stored in air, the activity was lost at all tested temperatures (−20, 4, and 25 °C) within a month (Figure 4a). When the powder was stored under oxygen-free conditions (Figure 4b), the activity was retained for much longer. The tendency of rAAT to undergo methionine oxidation, therefore, requires that both formulation and storage are carried out in the absence of oxygen.

#### 3.5.4. Formulation of hAAT

For comparison, the protocol set to produce the powder with rAAT was applied to a commercial preparation of hAAT, also containing NaHPO_4_ and NaCl (Prolastin^®^, Vicopisano, Italy). The freeze-dried product was reconstituted with ultrapure water to a final concentration of 0.5 mg/mL protein. Mannitol and L-leucine were added to obtain equal final concentrations. The solution was spray-dried, recovering 43% of the starting weight of the solid. The aerodynamic performances of the powder (SD-Prol) were assessed by an FSI, resulting in an emitted fraction of 60.86 ± 16.72% and a respirable dose of 3.35 ± 2.63 mg. The protein was fully soluble (Table 5), and its activity was satisfactorily preserved after spray drying (67%).

The possible aggregation of protein in the two rAAT formulations as well as the hAAT formulation was tested by dynamic laser scattering. The protein diameter obtained for rAAT after purification was 6.55 ± 1.4 nm, in good agreement with the computed value of the hydrodynamic diameter of a globular protein of about 50 kDa [45]. After dissolution of the formulated powders, protein diameters of 9.03 ±1.09, 17.31 ± 3.89, and 7.02 ± 1.12 nm were obtained for the mannitol, sucrose, and hAAt powders, respectively, the first and the latter being non-significantly different from the diameter of the non-formulated protein (*p* > 0.05).

## 4. Discussion

In this paper, a recombinant, non-glycosylated AAT was produced with a novel purification protocol at yields high enough for its formulation as powder and for preliminary in vitro experiments. Its functional properties and its formulation were compared with that of plasma-derived hAAT.

rAAT was fully functional in vitro, confirming previous observations that recombinant, non-glycosylated rAAT obtained in *E. coli* is indistinguishable from hAAT at inhibiting elastase [25]. rAAT was also very similar to hAAT in thermal stability (Figure 1c and Figure S2c), contrary to previous reports that showed a stabilizing effect exerted by glycosylation [46]. Crucially for the spray drying technique, rATT was only partially and reversibly denatured at temperatures up to 90 °C. Finally, a tendency to aggregation observed for non-glycosylated AATs [47] did not appear to be an issue for its formulation as a powder, and rAAT could be formulated similarly to hAAT. The absence of post-translational glycosylation can be pointed out as a critical aspect since it is reported to improve the stability and efficacy of augmentation therapy [46,48,49]. However, rAAT administration in mice by nasal instillation suggested that, differently from parenteral administration, the non-glycosylated forms of AAT could be even more protective than their glycosylated counterpart in the respiratory context [25]. These findings support a recombinant alternative to plasma-derived hAAT for the specific treatment of AATD-associated lung disease to prevent the risks associated with plasma manipulation and the variability deriving from naturally occurring isoforms in plasma pools from different donors, as well as from the manufacturing process [50], including the presence of plasma impurities.

Storage conditions of AAT solutions were shown to be critical for protein stability. Storage at temperatures above −80 °C resulted in a slow loss of activity (Figure 1d). Consistently, LM/MS analysis revealed a progressive mono- and di-oxidation of Met358 and Met351 (Figure S3). Storage and formulation under a controlled atmosphere slowed down the inactivation. This aspect, to date neglected in the literature, might help extend the shelf life of AAT-containing products.

Endotoxins were efficiently removed from the preparation (Figure S1). Bacterial endotoxin requirements are not regarded as a critical quality attribute in dry powder inhalants [51]. However, considering the recombinant source of the protein, as well as the potential lifelong administration of an AAT-based inhalable product, we considered this preliminary purification worthwhile, since inhaled endotoxins are known to cause airway and systemic side effects [52,53].

A screening of excipients was preliminarily performed under conditions known to be compatible with AAT stability and selecting salts, sugars, polyalcohols, and amino acids typically used in formulations for lung delivery [54]. Hygroscopic substances, such as sodium salts, known to reduce protein stability in powders, were reduced as much as possible. Potassium phosphate at 10 mM concentration was identified as a good compromise to allow protein solubility and its formulation. Three non-reducing bulking agents commonly used for the stabilization of proteins in spray drying, i.e., trehalose, mannitol, and sucrose [55], were tested. Trehalose was excluded since, in the selected drying conditions, it resulted in a very sticky powder. This behavior has already been reported [56,57] and might be due to the relatively low glass transition temperature (T_g_) of this sugar, reported to be between 72 °C and 120 °C [58] and measured as 80 °C in spray drying conditions similar to those used in this work [57]. Moreover, the T_g_ of trehalose could be further reduced by its interaction with phosphate ions in solution [59]. For this reason, this excipient was excluded, despite its use as stabilizing agent reported in the literature for a similar formulation [19,22].

Mannitol and sucrose were further tested in association with amino acids L-leucine, L-lysine, and L-cysteine, which were expected to improve powder flowability, reduce hygroscopicity, and, for L-cysteine, to help prevent protein oxidation. No powder could be recovered when L-lysine was used, while L-cysteine led to low yields when combined with sucrose, or to a wet powder when combined with mannitol. Therefore, L-leucine, in combination with mannitol and sucrose, was selected as the most promising component to optimize the process parameters. A 0.7 mm nozzle led to good yields, with all powders showing very good aerodynamic performances in vitro, both in terms of emitted fraction and fine particle fraction (Table 2). The thermal analysis suggested that powders were in an amorphous state. This might be due to the low concentration of solids in the solution, which prevented the attainment of critical supersaturation in the evaporation process [60]. It is likely that the presence of L-leucine at a high weight ratio to sucrose or mannitol (1:1 or 2:1) and the presence of phosphates [61] were responsible for preventing their crystallization, which was considered useful in view of adding the protein, since excipients in the amorphous state are expected to provide better stabilization of spray-dried proteins [62]. Interestingly, a higher concentration of L-leucine did not significantly improve the yield, indicating that the concentration of excipients, and in particular L-leucine, could be fixed at the minimum tested level, 0.5 mg/mL, the same as mannitol, sucrose, and rAAT. Despite the possible humidity-mediated powder sticking due to the presence of phosphates, leucine relative weight (16.3% *w*/*w*) was not further increased, given the good aerodynamic characteristics obtained, and also because concentrations of leucine above 20% *w*/*w* were reported not to provide significant improvement in surface moisture protection and particle separation [63]. The thermogravimetric analysis confirmed that limited residual moisture was present in the formulation prepared from sucrose-based solutions, while the powder prepared with mannitol was completely dry. This result is remarkable since residual water might improve the molecular mobility of mannitol and could determine an undesired inactivation of rAAT. Both bulking agents were tested for the formulation of the protein.

When rAAT was formulated in the selected conditions, unexpectedly, the yield was reduced in comparison to protein-free preparations (Table 4). This might be due to the lower volumes of solution that were dried. In general, in the presence of rAAT, the aerodynamic behavior of powders worsened in comparison to the protein-free formulations, especially when sucrose was used as an excipient. SEM analysis (Figure 2) revealed the presence of larger aggregates in all powders containing rAAT with respect to the counterparts without protein, which could at least partially explain their worst aerodynamic performances. When analyzing the state of the protein, mannitol was effective at preventing its aggregation, since the diameter of the protein (both recombinant and human) after powder redissolution was not statistically different from that measured on the purified, non-formulated protein. This was not true for the sucrose-based formulation that, in addition, revealed a remarkable drop in antielastase activity. The weight ratio between bulking agent and protein is close to the values suggested by Schüle et al. [60] as optimal to preserve the stability of a spray-dried IgG1. On the other hand, sucrose is reported to be rather hygroscopic, as also observed by thermogravimetric analysis on powders (water content between 0.5 and 1.6%), and this could have negatively affected protein stability and activity. It is worth underlining that the same conditions of formulation, applied to hAAT, resulted in comparable results in terms of production, aerodynamic, and stability performances of powder with respect to rAAT (Table 5), indicating that glycosylation does not play a crucial role in these characteristics. In comparison to the formulation reported by Bar (EP3565584) [22], who reported data relevant to the production of human glycosylated AAT as a spray-dried powder, the mannitol-containing powder developed in the present work showed comparable aerodynamic performance, better (lower) residual moisture, and significantly lower activity. It is, however, worth underscoring that the activities were determined with different methods.

Considering the better performances in terms of yield, aerodynamic performances, and preservation of rAAT activity, mannitol was selected as a bulking agent, and the stability of the powder was assessed over time. When stored at −80 °C for two weeks, the powder did not show a significant increase in particle size (Figure 3), suggesting that no aggregation occurred due to the formation of interactions or water absorption. Crucially, we showed that protein stability in the powder required storage under oxygen-free conditions. Considering the LC/MS data on the protein solution, the loss of activity can be attributed to methionine oxidation. This represents an issue that needs consideration when producing an inhalable powder of AAT. In fact, previous reports on drying of AAT [19,20] introduced different antioxidants in the powder components. However, considering the expected life-long treatment with AAT, the presence of an antioxidant in the formulation may represent a toxicological concern. Therefore, the production of a preservative-free formulation, though less stable, should be considered an acceptable compromise in the first instance.

The most promising formulation contains an amount of active rAAT compatible with administration in patients. Indeed, augmentation therapy by parenteral infusion aims at reaching a serum concentration of at least 11 μM, which is estimated to correspond to a concentration in epithelial lung fluid (ELF) of 1.7 μM (8.8 mg/dL) [64]. Considering that the volume of ELF is estimated at a maximum of about 20 mL [65], at least 1.8 mg of fully active protein should reach the deep airways to achieve the desired concentration. To bring about said concentration with the formulation described in this work, about 50 mg of powder should be administered, for example, by discharging two size-3 capsules, each containing 25 mg of the formulation. This could constitute a convenient way for administering AAT to patients without the need for healthcare assistance and weekly hospitalization, as required for parenteral augmentation therapy.

## 5. Conclusions

Recombinant AAT was successfully produced and purified. The protein was fully active at inhibiting elastase, and endotoxins could be efficiently removed. A formulation with L-leucine and mannitol as excipients yielded a dry powder for inhalation, with good aerodynamic performances in vitro, suggesting acceptable respirability. The stability of the powder was assessed, defining a possible shelf-life and storage conditions. Antielastase activity was maintained upon formulation when stored at −20 °C under oxygen-free conditions. Far from being the ultimate formulative approach for rAAT as inhalable dry powder, the formulation described here concretely contributes to the development of a stable and highly respirable rAAT powder, which may represent an important tool to increase the efficacy of therapy, patient compliance, and improve the quality of life of patients suffering from alpha-1 antitrypsin deficiency.

## Figures and Tables

**Table 1:** Coding of variables for the 2^3^ full factorial design.

Variable	−1	+1
Bulking agent	Sucrose	Mannitol
Leucine concentration (mg/mL)	0.5	1
Nozzle diameter (mm)	0.5	0.7

**Table 2:** Experimental matrix, yield, aerodynamic parameters, and dynamic angle of repose (DAR) of powders prepared from solutions of L-leucine and sucrose or mannitol as bulking agents.

Sample ID	Input Parameters			Output			
	x_1_ = Type of Bulking Agent	x_2_ = Leucine Concentration (mg/mL)	x_3_ = Nozzle Diameter (mm)	Yield (%)	Emitted Fraction (%)	Fine Particle Fraction (%)	DAR (°)
S-0.5-0.5	Sucrose	0.5	0.5	46.66	84.4 ± 1.47	84.2 ± 17.2	42° ± 1°
S-0.5-0.7			0.7	72.19	85.07 ± 2.63	64.8 ± 9.9	37° ± 3°
S-1-0.5		1	0.5	54.4	81.25 ± 3.30	90.7 ± 7.6	45 ° ± 1°
S-1-0.7			0.7	76.32	81.84 ± 2.61	72.9 ± 6.4	36° ± 3°
M-0.5-0.5	Mannitol	0.5	0.5	62.88	84.89 ± 1.07	86.3 ± 16.3	37° ± 2°
M-0.5-0.7			0.7	73.41	91.24 ± 9.58	78.8 ± 7.2	38° ± 1°
M-1-0.5		1	0.5	69.89	78.43 ± 3.17	92.8 ± 10.8	47° ± 1°
M-1-0.7			0.7	72.27	98.56 ± 0.65	67.2 ± 0.2	37° ± 11°

**Table 3:** Particle size distribution data of different excipient mixtures.

Formulation	Dv_10_ μm	Dv_50_ μm	Dv_90_ μm	Span
S-0.5-0.5	1.15	2.34	5.49	1.85
S-0.5-0.7	2.05	4.49	8.69	1.48
S-1-0.5	1.04	1.98	8.94	3.99
S-1-0.7	2.63	4.34	6.77	0.94
M-0.5-0.5	1.06	2.12	5.44	2.07
M-0.5-0.7	1.46	2.74	5.18	1.36
M-1-0.5	1.06	2.12	5.44	2.07
M-1-0.7	1.64	3.47	7.54	1.70

**Table 4:** Characteristics of powders prepared from 50 mL solutions of rAAT with L-leucine and sucrose or mannitol as bulking agents.

Bulking Agent	Yield (%)	Emitted Fraction (%)	Fine Particle Fraction (%)	AAT Soluble Fraction	AAT Activity
Sucrose	40.65	61.8 ± 3.4	39.2 ± 6.3	100%	18%
Mannitol	35.18	87.3 ± 0.4	41.54 ± 14.1	100%	54%

**Table 5:** Characteristics of the powder prepared from solutions of plasma-derived AAT with L-leucine and mannitol as a bulking agent.

Formulation	Yield (%)	Emitted Fraction (%)	Fine Particle Fraction (%)	AAT Soluble Fraction	AAT Activity on Elastase
SD-Prol	43.44	60.9 ± 16.7	32.34 ± 25.39	100%	67%

## Data Availability

Data are contained within the article or Supplementary Material.

## Supplementary Materials

The following supporting information can be downloaded at: https://www.mdpi.com/article/10.3390/pharmaceutics14122754/s1, Figure S1: Proinflammatory activity of rAAT before and after treatment with Sartobind Q STIC PA capsules for endotoxin removal; Figure S2: Characterization of hAAT. Figure S3: Mass spectrometry of rAAT upon storage; Figure S4: Solubility of rAAT in solutions at different concentrations of potassium phosphate.
