# Supplementary material for: Recombinant Alpha-1 Antitrypsin as Dry Powder for Pulmonary Administration: A Formulative Proof of Concept
Source: Pharmaceutics. 2022 Dec 8;14(12):2754. doi: 10.3390/pharmaceutics14122754 (PMC9784676; doi:10.3390/pharmaceutics14122754)
Supplement: Supplementary file 1 [file pharmaceutics-14-02754-s001.zip › pharmaceutics-2044922-supplementary.pdf]

Supplementary data

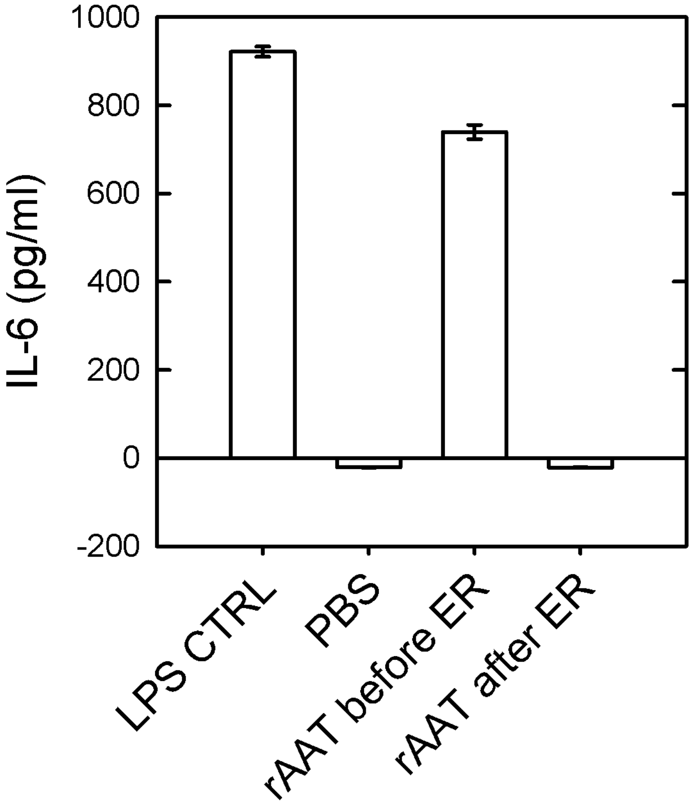

Figure S1 Proinflammatory activity of rAAT before and after treatment with Sartobind Q STIC PA capsules for endotoxin removal (ER). Human macrophages were incubated with the PBS solution used for rAAT dilution (PBS), with the rAAT solution 1.8 µg/ml before and after ER, and, also with a lipopolysaccharide 1µg/ml solution (LPS CTRL) as a positive control. No IL-6 was detected in cell supernatants upon incubation with either PBS or powder rAAT preparations after ER. Bars represent standard deviation of triplicate analyses. PBS and rAAT after ER vs LPS CTRL $p<0.0001$ (Anova with Tukey post-hoc analysis).

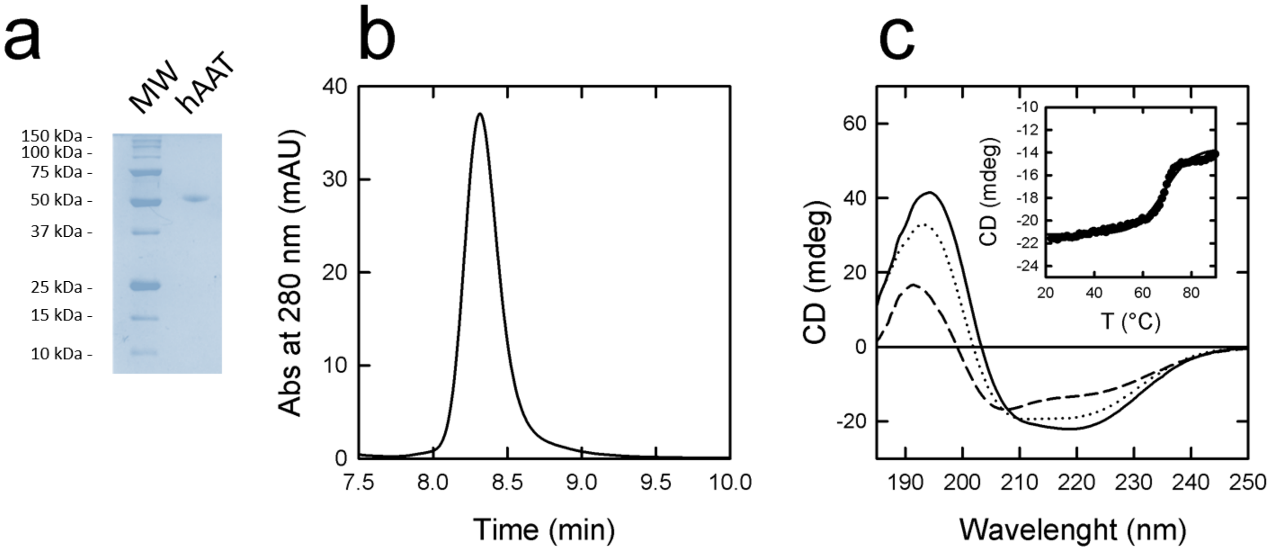

Figure S2: **Characterization of hAAT.** (a) Coomassie blue-stained SDS-PAGE gels of purified hAAT. (b) SEC chromatogram (c) Circular dichroism spectra of hATT (solid line), hAAT upon incubation at 90°C (dashed line) and after incubation at 90°C and slow return to 20 °C (dotted line). Inset: temperature ramp, with a calculated Tm of 69 °C.

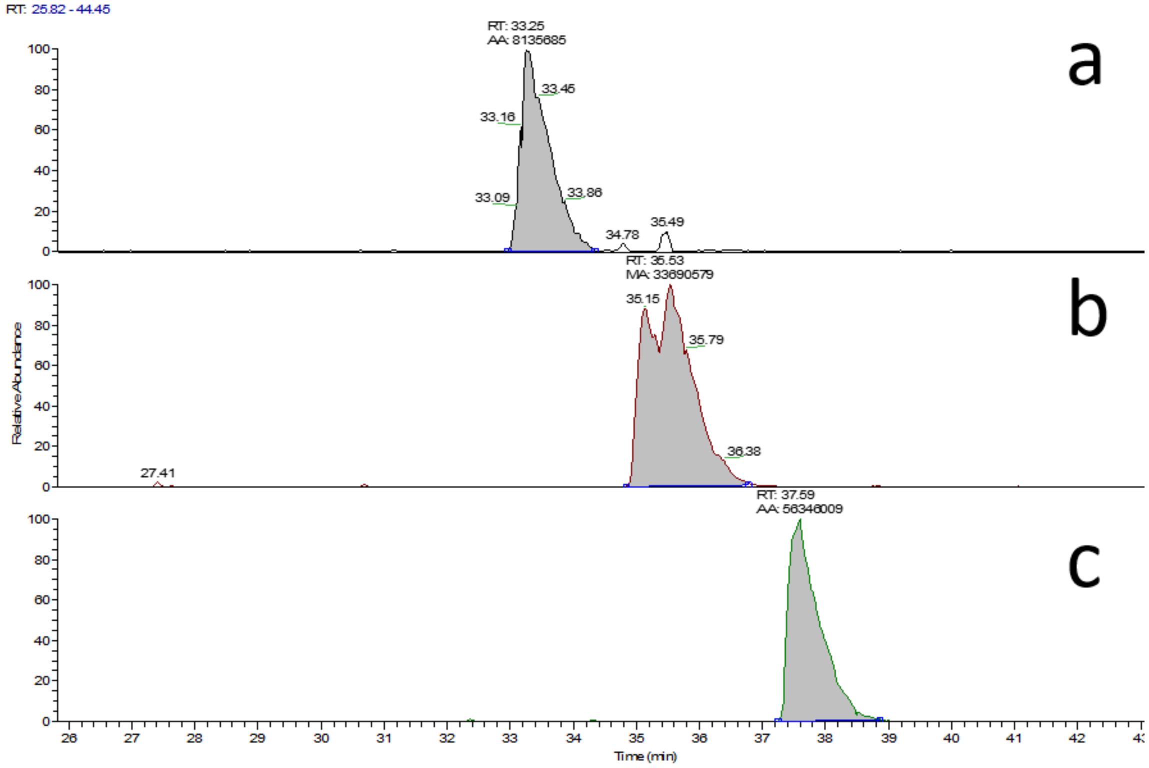

Figure S3: Mass spectrometry of rAAT upon storage. Semi-quantitative LC-MS analysis of peptide KGTEAAGAMFLEAIPMSIPPEVKF (2259.03 Da) containing both Met358 and Met351: **a.** Triple charged of di-oxidized form; **b.** Triple charged of mono-oxidized form; **c.** Triple charged of non-oxidized form.

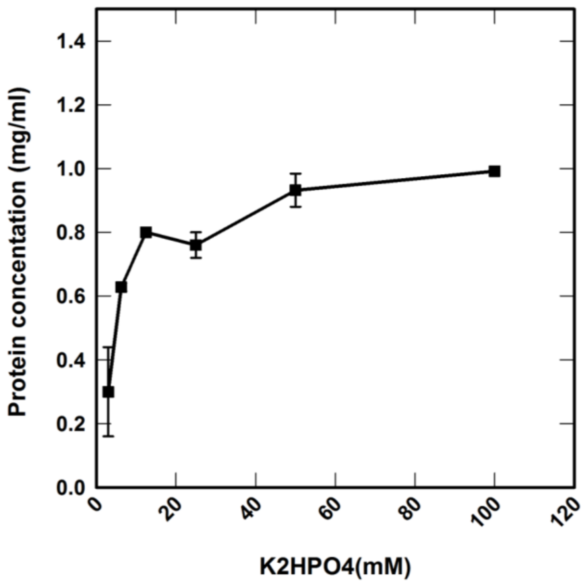

Figure S4: Solubility of rAAT in solutions at different concentrations of potassium phosphate. The protein at 1 mg/mL concentration, originally in PBS buffer, was dialyzed against phosphate buffers, and the soluble fraction upon centrifugation was estimated by SDS-PAGE.
